# Overcoming the problem of multicollinearity in sports performance data: A novel application of partial least squares correlation analysis

**DOI:** 10.1371/journal.pone.0211776

**Published:** 2019-02-14

**Authors:** Dan Weaving, Ben Jones, Matt Ireton, Sarah Whitehead, Kevin Till, Clive B. Beggs

**Affiliations:** 1 Institute for Sport, Physical Activity and Leisure, Leeds Beckett University, Leeds, West Yorkshire, United Kingdom; 2 Leeds Rhinos Rugby League club, Leeds, United Kingdom; 3 Yorkshire Carnegie Rugby Union club, Leeds, United Kingdom; 4 The Rugby Football League, Leeds, United Kingdom; 5 Warrington Wolves Rugby League club, Warrington, United Kingdom; University of Pittsburgh, UNITED STATES

## Abstract

**Objectives:**

Professional sporting organisations invest considerable resources collecting and analysing data in order to better understand the factors that influence performance. Recent advances in non-invasive technologies, such as global positioning systems (GPS), mean that large volumes of data are now readily available to coaches and sport scientists. However analysing such data can be challenging, particularly when sample sizes are small and data sets contain multiple highly correlated variables, as is often the case in a sporting context. Multicollinearity in particular, if not treated appropriately, can be problematic and might lead to erroneous conclusions. In this paper we present a novel ‘leave one variable out’ (LOVO) partial least squares correlation analysis (PLSCA) methodology, designed to overcome the problem of multicollinearity, and show how this can be used to identify the training load (TL) variables that influence most ‘end fitness’ in young rugby league players.

**Methods:**

The accumulated TL of sixteen male professional youth rugby league players (17.7 ± 0.9 years) was quantified via GPS, a micro-electrical-mechanical-system (MEMS), and players’ session-rating-of-perceived-exertion (sRPE) over a 6-week pre-season training period. Immediately prior to and following this training period, participants undertook a 30–15 intermittent fitness test (30-15_IFT_), which was used to determine a players ‘starting fitness’ and ‘end fitness’. In total twelve TL variables were collected, and these along with ‘starting fitness’ as a covariate were regressed against ‘end fitness’. However, considerable multicollinearity in the data (VIF >1000 for nine variables) meant that the multiple linear regression (MLR) process was unstable and so we developed a novel LOVO PLSCA adaptation to quantify the relative importance of the predictor variables and thus minimise multicollinearity issues. As such, the LOVO PLSCA was used as a tool to inform and refine the MLR process.

**Results:**

The LOVO PLSCA identified the distance accumulated at very-high speed (>7 m·s^-1^) as being the most important TL variable to influence improvement in player fitness, with this variable causing the largest decrease in singular value inertia (5.93). When included in a refined linear regression model, this variable, along with ‘starting fitness’ as a covariate, explained 73% of the variance in v30-15_IFT_ ‘end fitness’ (p<0.001) and eliminated completely any multicollinearity issues.

**Conclusions:**

The LOVO PLSCA technique appears to be a useful tool for evaluating the relative importance of predictor variables in data sets that exhibit considerable multicollinearity. When used as a filtering tool, LOVO PLSCA produced a MLR model that demonstrated a significant relationship between ‘end fitness’ and the predictor variable ‘accumulated distance at very-high speed’ when ‘starting fitness’ was included as a covariate. As such, LOVO PLSCA may be a useful tool for sport scientists and coaches seeking to analyse data sets obtained using GPS and MEMS technologies.

## Introduction

Professional sporting organisations invest considerable resources collecting and analysing data to better understand the factors that influence athletic performance. Recent advances in wearable technology and computing power mean that large volumes of data are now readily available to the applied practitioner [1]. However, while this data is becoming easier to collect, analysing it can be a challenging task, particularly when sample sizes are small (i.e. limited by squad size) and the data is highly correlated–something that can lead to instability when applying standard least squares regression techniques, making it difficult to draw firm inference [2–3]. With respect to this, global positioning system (GPS) and micro-electrical-mechanical-system (MEMS) data can be particularly problematic [4–5]. GPS and MEMS are often used to measure an athlete’s movement, from which speed, distance travelled, and acceleration can be computed using standard mathematical algorithms. For example, a player’s velocity and acceleration are simply the first and second derivatives of the distance travelled. Consequently, these variables are not independent, but instead are highly correlated. It is therefore not surprising that strong correlations have been reported between variables widely used to assess training load (TL) [4–5].

Whilst individual measured variables are collected when acquiring performance data, these are often grouped together to represent latent constructs such as ‘fitness’, ‘fatigue’ or ‘technical-tactical performance’. For example, a rugby league coach might represent ‘attacking performance’ using variables such as the ‘number of metres run’, ‘tackle breaks’ or ‘kick return metres’ [6]. Conversely, a sports scientist might assess the TL imposed using data acquired from GPS or MEMS [4]. However, quantifying the relationships within and between these various latent constructs can be challenging because the individual variables used to quantify, for example, TL in athletes, have been shown in a meta-analysis to possess substantial correlations [5], making them prone to multicollinearity issues [3,5]. Nevertheless, when attempting to understand relationships between the latent constructs used in sports performance, multicollinearity issues are often ignored, with the tendency being instead to rely on univariate analysis [7–10]. However, univariate analysis has the inherent drawback that it assumes that the variables are independent and does not allow for any covariance within the data, something that can be a major weakness. Furthermore, it is not possible using univariate analysis to ‘capture’ any information that may be associated with the covariance between the variables, something that might be particularly important when considering the effect that changes in prescribed training mode across a training period might have on the relationships between TL variables [4–5, 11–12]. Consequently, when analysing TL data it is important to allow for covariance in the data in order to ensure that appropriate inference is drawn and that erroneous conclusions are not reached.

When more sophisticated analysis is used, multiple linear regression (MLR) is generally the tool used by practitioners to quantify the strength of relationships within TL data. It is common practice when performing MLR to retain only those predictor variables that ‘confidently explain’ the behaviour of the response (dependent) variable, with variables that fail to reach a required level of significance excluded. While this approach works well when the predictor variables are weakly correlated with each other, problems can occur when strong correlations are present and variable inflation factors (VIFs) are >10 [13–15]. Multicollinearity over-inflates the standard errors associated with the respective regression coefficients, causing the p-values to become very sensitive to changes in model specification, resulting in the whole process becoming unstable. Consequently, multiple competing models may be produced, making it difficult to be confident about any inference drawn from the various models [2]. In contrast to MLR, singular value decomposition (SVD) is immune to multicollinearity because it produces a set of orthogonal composite variables that are completely uncorrelated [16–18]. Although not a statistical technique *per se*, SVD underpins other techniques such as principal component analysis (PCA) and partial least squares correlation analysis (PLSCA) [19–20]. PLSCA in particular appears to have considerable potential with regard to the analysis of small data sets that exhibit multicollinearity (e.g. sports performance data sets). Because PLSCA incorporates SVD, it has the great advantage that it is both immune to multicollinearity, and unlike MLR, can cope with situations where the number of predictor variables exceeds the number of observations. Unlike MLR, which involves one response (dependent) variable and multiple predictor (independent) variables, PLSCA sub-divides the data into two blocks (sub-groups) each containing one or more variables, and then uses SVD to establish the strength of any relationship that might exist between the two component sub-groups [21]. It does this by using SVD to determine the inertia (sum of the singular values) of the covariance matrix of the sub-groups under consideration [17,19,21]. Given that the singular values are proportional to the magnitude of any effect [17], the higher the value of the singular value inertia observed, the greater the amount of shared information between the respective sub-groups and the stronger the relationship between the two. PLSCA is usually accompanied by a one-tailed permutation test generally involving 10,000 random permutations in order to establish the null distribution of the possible inertias and the likelihood (the odds) of the observed relationship occurring by chance [21].

Although PLSCA has been widely used in neuroimaging [17,19–20,22], it has not been used in sport science, with the result that its potential in this discipline has not been exploited. We therefore hypothesised that PLSCA might be a useful tool with which to analyse small sport performance data sets. As sport scientists regularly seek to identify the TL variables that best relate to training outcome measures, we designed the study presented here with the aim of investigating the relationship between the accumulation of TL (quantified using multiple collection methods), and 30–15 intermittent fitness test (30-15_IFT_) performance achieved by 16 professional youth rugby league players following a 6-week training period. The overall aim of the study was to establish whether or not PLSCA, coupled with a novel ‘leave one variable out (LOVO)’ adaptation, might be a useful tool for analysing TL data sets where multicollinearity issues are problematic. More specifically, we wanted to know if the above methodology could identify which accumulated TL variables best relate to 30-15_IFT_ performance following 6-weeks of training.

### Leave one variable out PLSCA example

In order to explain the linear algebra underpinning PLSCA and to highlight the LOVO adaptation, we shall first consider a small dataset (Table 1) containing publicly available data collected from the twelve teams competing in the European Super League during the 2017 season (https://rugby-league.com/superleague/stats/club_stats). In this data set we have two outcome variables for the season: total league points accumulated and total match score difference; and three performance related variables: the number of missed tackles; the number of tackle busts; and the number of clean breaks. If for example, we wanted to establish whether or not a relationship exists between these two groups of variables, we could perform PLSCA to assess the amount of shared information common to the two and use this to quantify the strength of any relationship.

**Table 1:** League outcome and match performance data for the teams in the European Super League (season 2017).

Team	League Points	Score difference	Number of missed tackles	Number of tackle busts	Number of clean breaks
Castleford Tigers	40	391	1523	918	208
Leeds Rhinos	30	76	1588	796	153
Wigan Warriors	23	21	1574	697	157
Warrington Wolves	20	-145	1654	830	165
Wakefield Trinity Wildcats	26	63	1519	698	174
Salford Red Devils	26	76	1525	784	178
Huddersfield Giants	21	35	1656	757	143
Hull FC	27	27	1522	872	180
St Helens	25	25	1746	744	166
Leigh Centurions	12	12	1639	606	159
Widnes Vikings	11	-269	1652	626	136
Catalans Dragons	15	-220	1451	701	129

PLSCA is generally performed by first mean centring and standardizing the data to unit variance [23] and then dividing it into two matrices, in this case a [12×2] matrix, *X*, containing the variables total league points accumulated and total score difference, and a [12×3] matrix, *Y*, containing the number of missed tackles, the number of tackle busts, and the number of clean breaks. The pattern of relationships between the columns of *X* and *Y* can be stored in a [3×2] covariance matrix, *R*, [21] which is computed as follows:

$$R=Y^{T}X={\left[\begin{array}{lll} -0.770 & 1.765 & 2.200\\ 0.007 & 0.465 & -0.433\\ -0.161 & -0.591 & -0.248\\ 0.796 & 0.827 & 0.124\\ -0.818 & -0.580 & 0.542\\ -0.746 & 0.337 & 0.727\\ 0.820 & 0.049 & -0.898\\ -0.782 & 1.275 & 0.820\\ 1.897 & -0.090 & 0.170\\ 0.617 & -1.561 & -0.155\\ 0.772 & -1.348 & -1.222\\ -1.631 & -0.548 & -1.547 \end{array}\right]}^{T}\times \left[\begin{array}{cc} 2.104 & 2.274\\ 0.866 & 0.405\\ 0.000 & 0.079\\ -0.371 & -0.906\\ 0.371 & 0.328\\ 0.371 & 0.405\\ -0.248 & 0.162\\ 0.495 & 0.115\\ 0.248 & 0.162\\ 0.495 & 0.115\\ 0.248 & 0.103\\ -1.362 & 0.026 \end{array}\right]=\left[\begin{array}{cc} -2.984 & -1.864\\ 8.985 & 6.408\\ 8.739 & 9.043 \end{array}\right]$$ (1)

SVD of the matrix, *R*, yields three orthogonal matrices: a [3×2] left singular vector matrix, *U*, containing the saliences (weights) for matrix, *Y*; a [2×2] right singular vector matrix, *V*, containing the saliences (weights) for matrix, *X*; and *S*, a [2×2] diagonal matrix containing the singular values [21].

$$R=USV^{T}=\left[\begin{array}{cc} -0.204 & 0.342\\ 0.645 & -0.658\\ 0.736 & 0.671 \end{array}\right]\times \left[\begin{array}{cc} 17.021 & 0.000\\ 0.000 & 1.617 \end{array}\right]\times {\left[\begin{array}{cc} 0.754 & -0.656\\ 0.656 & 0.754 \end{array}\right]}^{T}$$ (2)

The quantity of common information shared between *X* and *Y* can be directly quantified by computing the inertia, *ø*, (i.e. the sum of all the non-zero singular values) common to the two sub-groups, as follows [21]:

$$\varphi =\sum_{i}^{n}s_{i}=17.021+1.617=18.638$$ (3)

where; *n* is the number of non-zero singular values of matrix *R*; and *s*_*i*_ is *i*^*th*^ diagonal element of matrix *S*. Singular value inertia is an effect size, which directly relates to the strength of the relationship between the two sub-groups *X* and *Y* (i.e. the greater the magnitude of the inertia to more shared information common to *X* and *Y*).

**Fig 1:**
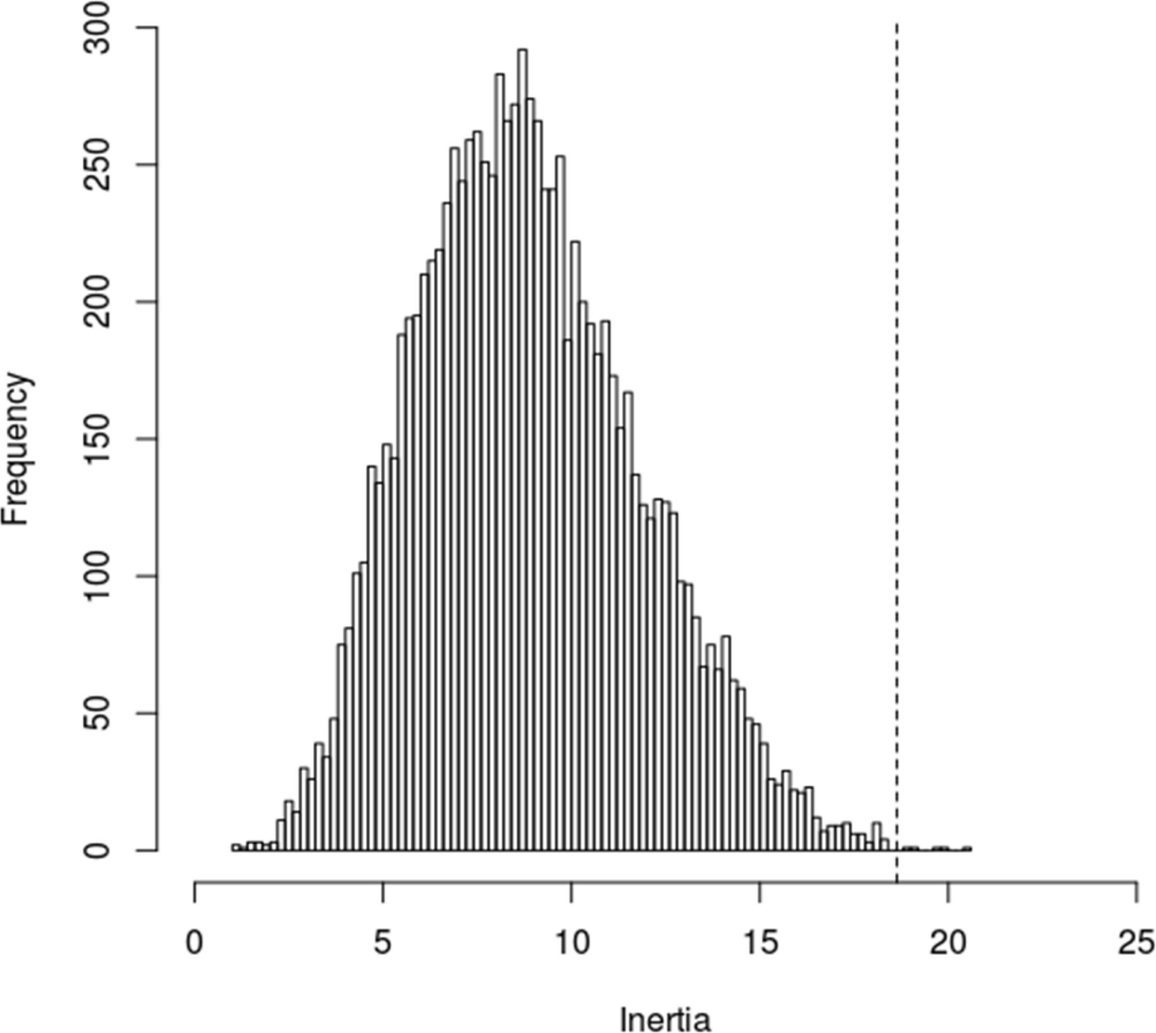
Singular value inertial value (indicated by dotted line) computed from the observed data and the null-distribution of the inertia computed using a permutation test with 10,000 permutations.

The statistical significance of the calculated inertia value can then be assessed using a permutation test in which the rows of *Y* are randomly permutated 10,000 times [21], to produce the null distribution of all the possible inertia values that could occur just by chance, as shown in Fig 1. From this it can be seen that an inertia value of ≥18.638 only occurred 11 times in 10,000 simulations. This equates to a p-value of 0.0011 and indicates that, perhaps unsurprisingly, a very strong relationship exists between the match performance and season outcome variables, something that is unlikely to have occurred by chance.

Having established that a strong relationship exists between the performance variables and season outcome, it is possible to interrogate the data further to identify which variables are most influential. In order to do this in a systematic manner, we developed a novel LOVO strategy, mirroring similar approaches adopted for SVD [18] and random forests [23], which involved repeating the PLSCA several times, with a different *Y* variable excluded from the analysis on each occasion. By observing the effect of each successive omission on the magnitude of the inertia, it is possible to assess the relative contribution of each performance variable to league outcome. In the example above, when the ‘number of missed tackles’ is omitted from the analysis, the inertia falls only marginally to 18.178, suggesting that this variable is not particularly influential. However by comparison, when the ‘number of tackle busts’ and the ‘number of clean breaks’ are omitted, then the inertia falls to 13.854 and 11.786 respectively, indicating that these two variables are much stronger predictors of league outcome.

## Materials and methods

### Participants

An observational research study was conducted in which the accumulated TL of sixteen male professional youth rugby league players (age [y]: 17.7 ± 0.9; height [cm]: 179.6 ± 5.5; body mass [kg]: 87.0 ± 8.8) was quantified via GPS, MEMS and session-rating-of-perceived-exertion (sRPE) during a 6-week pre-season training period. Immediately prior to and following this training period, participants undertook the 30–15 intermittent fitness test (30-15_IFT_), which was used to determine a players ‘starting fitness’ and ‘end fitness’. The content of the training and testing periods was prescribed by the coaching staff and included 3 to 4 field-based sessions per week comprising technical-, tactical-, sprint-, interval- and small-sided-games-based-training. The total number of recorded field training sessions was 273 with players participating in 17 ± 3 sessions. All procedures performed in the study were in accordance with the Leeds Beckett University ethics board which approved of the study prior to data collection and which conformed to the 1964 Helsinki Declaration. Informed consent was obtained from all participants prior to data collection.

### Procedures

The 30-15_IFT_ was conducted on artificial turf following two days of complete rest and prior to any additional training as per previous methods [24]. Players possessed familiarity with the 30-15_IFT_ as part of their regular monitoring practices. The 30-15_IFT_ comprised 30 second shuttles run over 40m, with 15 seconds of recovery. The speed of the test was controlled by an audible sound. At this time of the sound, players were required to be within a 3 m tolerance zone at either end or the middle of the 40 m shuttle. The start speed of the test was 8 km·h^-1^ and increased by 0.5 km·h^-1^ following each successive shuttle. The test terminated when players were no longer able to maintain the required speed of the test or when they did not reach the 3 m tolerance zone on three consecutive occasions. The last completed velocity during the test was taken as v30-15_IFT_. The v30-15_IFT_ achieved prior to the start of the training period was deemed to be each players ‘starting fitness’ whilst the v30-15_IFT_ achieved at the completion of the six-week training period was deemed to be each players ‘end fitness’.

All external training load measures were collected concurrently during each training session using 10 Hz GPS devices with in-built 100 Hz tri-axial accelerometer, gyroscope and magnetometer (Optimeye S5, Catapult Innovations, Melbourne, Victoria; firmware version: 7.17). This data was downloaded into specialist software (Catapult Sprint v5.1.7, Catapult Innovations, Melbourne, Victoria). The device was positioned between the scapulae within a manufacturer designed vest according to typical procedures. Each player wore the same unit for each session to limit potential between-unit variability in the data collected [25]. The mean number of satellites and horizontal dilution of precision (HDOP) during the data collection period was 15 ± 3 and 0.8 ± 0.6 respectively [25].

Derived from GPS, the total distance (m) covered during the ~17 training sessions was further differentiated into the distances (m) covered at arbitrary speed thresholds of low- (0 to 3 m∙s^-1^; SZ1), moderate- (3.1 to 5 m∙s^-1^; SVZ2), high- (5.1 to 7 m∙s^-1^; SZ3) and very-high-speeds (> 7.1 m∙s^-1^; SZ4). The minimum effort duration for each of the speed zones were set at 1 second [26]. An individualised high-speed threshold (IndSZ) was also calculated for each player, which was defined as the distance covered above the terminal speed achieved during the 30-15_IFT_ prior to commencement of the training programme. Between the players, this speed threshold ranged from 4.58 to 5.41 m·s^-1^.

Derived from the tri-axial accelerometer, total session PlayerLoad is a modified vector magnitude and is expressed as the square root of the sum of the squared instantaneous rate of change in acceleration in each of the three axes (X, Y, and Z) and divided by 100 [27]. This was further differentiated into four zones relating to low- (0 to 1 AU; PLZ1), moderate- (1.1 to 2 AU; PLZ2), high- (2.1 to 3 AU; PLZ3) and very-high (>3 AU; PLZ4) accumulation of PlayerLoad. All PlayerLoad variables were expressed in arbitrary units (AU). PlayerLoad has previously been shown to possess acceptable reliability [27]. sRPE was calculated for each player during the study period using the method of Foster et al. [28]. Exercise intensity for sRPE was determined using the Borg category ratio-10 scale, with players providing this ~15 to 30 minutes following the cessation of the session [28]. This was then multiplied by the training-session duration to calculate the sRPE training load in AU.

### Statistical analysis

In order to assess the strength of the relationships between the variables, we first undertook Pearson correlation analysis and then performed MLR analysis with ‘end fitness’ as the response variable and all the other variables included as predictors. VIF values were then calculated for each of the respective predictor variables, with those >10 identified as being particularly problematic [13–15].

In the study, PLSCA was used as a filtering tool to by-pass any multicollinearity problems and identify those variables that were most influential in predicting improvements in fitness. This involved performing a baseline PLSCA (as described above) with the variables divided into two groups: an ‘output’ sub-group containing the variables ‘starting fitness’ and ‘end fitness’, and a ‘predictor’ sub-group comprising all the TL variables. We included both the ‘fitness’ variables in the ‘output’ group, because collectively they contained more information about improvements in fitness than would have been the case if just the difference in the fitness level had been used. Once the baseline inertia (i.e. the calculated inertia with all the predictor variables included in the model) and its associated p value were calculated, the whole PLSCA process was then repeated with one predictor variable omitted from the analysis and the new inertia and p value noted. This process was repeated with a different predictor variable omitted each time (as described above), until the contribution of all the variables had been evaluated individually. Having done this, those variables that were deemed influential were used to construct refined PLSCA and MLR models. Improvements derived from refining the baseline PLSCA model were assessed using the Chi- square test and Cramer’s V. MLR analysis was performed with 'end fitness' as the response variable and 'starting fitness' as a covariate, as recommended by Allison [29]. All analysis was undertaken using a combination of in-house algorithms written in Matlab (version R2016b: utilising the ‘Statistics and machine learning’ toolbox) (Math-Works, Natick, MA) and R (version 3.3.2: utilising the packages: ‘psych’; ‘car’; and ‘pracma’) (open source software). For all analyses, p values <0.05 were deemed significant.

## Results

The TL descriptive results are presented in Table 2 along with the study data collected for each of the 16 subjects.

**Table 2:** Training load (TL) data and termination speed during 30–15 intermittent fitness test for 16 professional youth rugby league players.

Player ID	TD	SZ1	SZ2	SZ3	SZ4	PL	sRPE	IndSZ	PLZ1	PLZ2	PLZ3	PLZ4	Start Fitness	End Fitness
1	53844	36323	14962	2196	358	7115	16027	4674	1985	3504	1269	358	16.5	18.0
2	70550	43477	22926	3682	464	8241	18848	7072	2471	4569	1051	151	16.5	17.5
3	55967	35915	16397	3376	278	5665	11952	4521	2335	2516	544	270	17.0	17.5
4	57847	35950	18612	2713	535	5798	17276	3960	2168	2806	522	270	17.0	19.0
5	42585	28529	11444	2260	352	4453	12475	3950	2001	1932	360	161	17.0	18.0
6	63157	41285	17447	3876	520	6492	15594	4712	2699	3126	530	138	17.5	19.5
7	63540	40009	18602	4227	699	6394	14806	5453	2665	2819	673	238	17.5	20.0
8	63833	41708	18462	3090	567	6955	13744	4180	2692	3484	630	150	17.5	19.0
9	47832	29853	13184	3885	897	4988	12059	4379	2145	2353	381	109	17.5	19.0
10	67531	42024	20176	4491	840	7221	15251	5152	2805	3215	845	356	18.5	19.5
11	54425	34689	16342	2911	483	5655	15039	2870	2270	2766	460	160	18.5	19.0
12	62172	39659	17703	4094	705	6072	16563	4574	2766	2670	514	123	18.5	20.0
13	76006	45183	23589	6133	1100	8041	13024	8178	2688	3776	1157	421	19.0	20.5
14	35828	21557	10290	2989	992	3524	14590	2982	1362	1601	318	242	19.0	20.5
15	52281	33999	15164	2272	633	5928	14316	3279	2204	2956	568	200	19.5	19.5
16	47583	28391	15613	3154	425	4546	11090	2919	2102	1967	378	100	19.5	20.0
**Mean**	57186	36159	16932	3459	616	6068	14541	4553	2335	2879	638	215	17.9	19.2
**SD**	10579	6491	3626	1012	240	1294	2102	1442	386	750	292	98	1.0	1.0

Abbreviations: TD = total distance (m); SZ1 = speed zone 1 (0 to 3 m·s-1; [m]); SZ2 = speed zone 2 (3.1 to 5 m·s-1; [m]); SZ3 = speed zone 3 (5.1 to 7 m·s-1; [m]); SZ4 = speed zone 1 (> 7.1 m·s-1; [m]); PL = PlayerLoad (AU); PLZ1 = PlayerLoad Zone 1 (0 to 1 AU); PLZ2 = PlayerLoad Zone 2 (1.1 to 2 AU); PLZ3 = PlayerLoad Zone 3 (2.1 to 3 AU); PLZ4 = PlayerLoad Zone 4 (> 3.1 AU); sRPE = session-rating-of-perceived-exertion; IndSZ = Individualised speed zone (> 30–15 intermittent fitness test termination speed).

Pearson correlation analysis (Table 3) revealed multiple strong correlations between the predictor variables in the data, suggesting that the data exhibited considerable multicollinearity, something that was confirmed by the extremely high VIF values obtained when MLR analysis was performed (Table 4). From Table 4 it can be seen that all but one (i.e. sRPE) of the predictor variables exhibited a VIF>10, with most having values >1000. However, despite numerous strong relationships in the data, ‘end fitness’ was only significantly positively correlated with the variables ‘SZ4’ (r = 0.738, p = 0.001) and ‘starting fitness’ (r = 0.784, p<0.001) (Table 3).

**Table 3:** Results of the Pearson correlation analysis between training load variables, starting fitness and end fitness.

	TD	SZ1	SZ2	SZ3	SZ4	PL	sRPE	IndSZ	PLZ1	PLZ2	PLZ3	PLZ4	Starting Fitness	End Fitness
**TD (r value)**	**1.000**	**0.979**	**0.965**	**0.683**	**0.157**	**0.925**	**0.403**	**0.796**	**0.875**	**0.841**	**0.666**	**0.342**	**-0.140**	**0.022**
TD [p value]	NA	[0.000]	[0.000]	[0.004]	[0.560]	[0.000]	[0.122]	[0.000]	[0.000]	[0.000]	[0.005]	[0.195]	[0.605]	[0.937]
**SZ1 (r value)**	**0.979**	**1.000**	**0.907**	**0.569**	**0.038**	**0.938**	**0.433**	**0.738**	**0.895**	**0.857**	**0.668**	**0.307**	**-0.239**	**-0.079**
SZ1 [p value]	[0.000]	NA	[0.000]	[0.021]	[0.889]	[0.000]	[0.094]	[0.001]	[0.000]	[0.000]	[0.005]	[0.248]	[0.372]	[0.771]
**SZ2 (r value)**	**0.965**	**0.907**	**1.000**	**0.656**	**0.143**	**0.882**	**0.414**	**0.775**	**0.776**	**0.824**	**0.648**	**0.335**	**-0.083**	**0.026**
SZ2 [p value]	[0.000]	[0.000]	NA	[0.006]	[0.597]	[0.000]	[0.111]	[0.000]	[0.000]	[0.000]	[0.007]	[0.205]	[0.761]	[0.922]
**SZ3 (r value)**	**0.683**	**0.569**	**0.656**	**1.000**	**0.651**	**0.483**	**-0.044**	**0.749**	**0.616**	**0.341**	**0.345**	**0.335**	**0.226**	**0.457**
SZ3 [p value]	[0.004]	[0.021]	[0.006]	NA	[0.006]	[0.058]	[0.872]	[0.001]	[0.011]	[0.197]	[0.191]	[0.205]	[0.401]	[0.075]
**SZ4 (r value)**	**0.157**	**0.038**	**0.143**	**0.651**	**1.000**	**0.048**	**-0.048**	**0.317**	**0.066**	**-0.017**	**0.065**	**0.309**	**0.511**	**0.738**
SZ4 [p value]	[0.560]	[0.889]	[0.597]	[0.006]	NA	[0.860]	[0.860]	[0.232]	[0.807]	[0.951]	[0.810]	[0.244]	[0.043]	[0.001]
**PL (r value)**	**0.925**	**0.938**	**0.882**	**0.483**	**0.048**	**1.000**	**0.487**	**0.792**	**0.722**	**0.965**	**0.858**	**0.418**	**-0.278**	**-0.180**
PL [p value]	[0.000]	[0.000]	[0.000]	[0.058]	[0.860]	NA	[0.056]	[0.000]	[0.002]	[0.000]	[0.000]	[0.108]	[0.296]	[0.505]
**sRPE (r value)**	**0.403**	**0.433**	**0.414**	**-0.044**	**-0.048**	**0.487**	**1.000**	**0.261**	**0.195**	**0.568**	**0.399**	**0.088**	**-0.347**	**-0.149**
sRPE [p value]	[0.122]	[0.094]	[0.111]	[0.872]	[0.860]	[0.056]	NA	[0.329]	[0.468]	[0.022]	[0.126]	[0.745]	[0.188]	[0.582]
**IndSZ (r value)**	**0.796**	**0.738**	**0.775**	**0.749**	**0.317**	**0.792**	**0.261**	**1.000**	**0.552**	**0.731**	**0.749**	**0.469**	**-0.264**	**-0.054**
IndSZ [p value]	[0.000]	[0.001]	[0.000]	[0.001]	[0.232]	[0.000]	[0.329]	NA	[0.027]	[0.001]	[0.001]	[0.067]	[0.323]	[0.843]
**PLZ1 (r value)**	**0.875**	**0.895**	**0.776**	**0.616**	**0.066**	**0.722**	**0.195**	**0.552**	**1.000**	**0.590**	**0.346**	**0.056**	**-0.060**	**0.076**
PLZ1 [p value]	[0.000]	[0.000]	[0.000]	[0.011]	[0.807]	[0.002]	[0.468]	[0.027]	NA	[0.016]	[0.190]	[0.835]	[0.824]	[0.780]
**PLZ2 (r value)**	**0.841**	**0.857**	**0.824**	**0.341**	**-0.017**	**0.965**	**0.568**	**0.731**	**0.590**	**1.000**	**0.828**	**0.301**	**-0.336**	**-0.281**
PLZ2 [p value]	[0.000]	[0.000]	[0.000]	[0.197]	[0.951]	[0.000]	[0.022]	[0.001]	[0.016]	NA	[0.000]	[0.257]	[0.204]	[0.292]
**PLZ3 (r value)**	**0.666**	**0.668**	**0.648**	**0.345**	**0.065**	**0.858**	**0.399**	**0.749**	**0.346**	**0.828**	**1.000**	**0.662**	**-0.280**	**-0.202**
PLZ3 [p value]	[0.005]	[0.005]	[0.007]	[0.191]	[0.810]	[0.000]	[0.126]	[0.001]	[0.190]	[0.000]	NA	[0.005]	[0.293]	[0.453]
**PLZ4 (r value)**	**0.342**	**0.307**	**0.335**	**0.335**	**0.309**	**0.418**	**0.088**	**0.469**	**0.056**	**0.301**	**0.662**	**1.000**	**-0.018**	**0.079**
PLZ4 [p value]	[0.195]	[0.248]	[0.205]	[0.205]	[0.244]	[0.108]	[0.745]	[0.067]	[0.835]	[0.257]	[0.005]	NA	[0.948]	[0.771]
**Starting Fitness (r value)**	**-0.140**	**-0.239**	**-0.083**	**0.226**	**0.511**	**-0.278**	**-0.347**	**-0.264**	**-0.060**	**-0.336**	**-0.280**	**-0.018**	**1.000**	**0.784**
Starting Fitness [p value]	[0.605]	[0.372]	[0.761]	[0.401]	[0.043]	[0.296]	[0.188]	[0.323]	[0.824]	[0.204]	[0.293]	[0.948]	NA	[0.000]
**End Fitness (r value)**	**0.022**	**-0.079**	**0.026**	**0.457**	**0.738**	**-0.180**	**-0.149**	**-0.054**	**0.076**	**-0.281**	**-0.202**	**0.079**	**0.784**	**1.000**
End Fitness [p value]	[0.937]	[0.771]	[0.922]	[0.075]	[0.001]	[0.505]	[0.582]	[0.843]	[0.780]	[0.292]	[0.453]	[0.771]	[0.000]	NA

**Abbreviations**: TD = total distance (m); SZ1 = speed zone 1 (0 to 3 m·s^-1^; [m]); SZ2 = speed zone 2 (3.1 to 5 m·s^-1^; [m]); SZ3 = speed zone 3 (5.1 to 7 m·s^-1^; [m]); SZ4 = speed zone 1 (> 7.1 m·s^-1^; [m]); PL = PlayerLoad (AU); PLZ1 = PlayerLoad Zone 1 (0 to 1 AU); PLZ2 = PlayerLoad Zone 2 (1.1 to 2 AU); PLZ3 = PlayerLoad Zone 3 (2.1 to 3 AU); PLZ4 = PlayerLoad Zone 4 (> 3.1 AU); sRPE = session-rating-of-perceived-exertion; IndSZ = Individualised speed zone (> 30–15 intermittent fitness test termination speed).

**Table 4:** Baseline multiple linear regression model with end fitness as the response variable, showing the calculated variable inflation factors (VIFs).

Response Variable	Predictor Variables	Coefficient (b)	Significance (p value)	VIF	Model Metrics Adj r2 (p value)
End Fitness	Intercept	1.289e+01	0.426	NA	0.562 (0.324)
	TD	1.322e-03	0.876	224288.3	
	SZ1	-8.480e-04	0.920	83232.3	
	SZ2	-1.172e-03	0.886	24699.2	
	SZ3	-1.529e-03	0.853	1930.9	
	SZ4	2.432e-03	0.765	104.0	
	PL	-1.206e-03	0.985	203338.7	
	sRPE	-3.599e-05	0.823	3.2	
	IndSZ	-2.923e-04	0.670	26.1	
	PLZ1	-3.033e-03	0.962	17170.2	
	PLZ2	-2.006e-03	0.975	65129.4	
	PLZ3	4.597e-03	0.945	10600.5	
	PLZ4	-7.791e-03	0.905	1131.4	
	Starting Fitness	3.200e-01	0.691	18.0	

**Fig 2:**
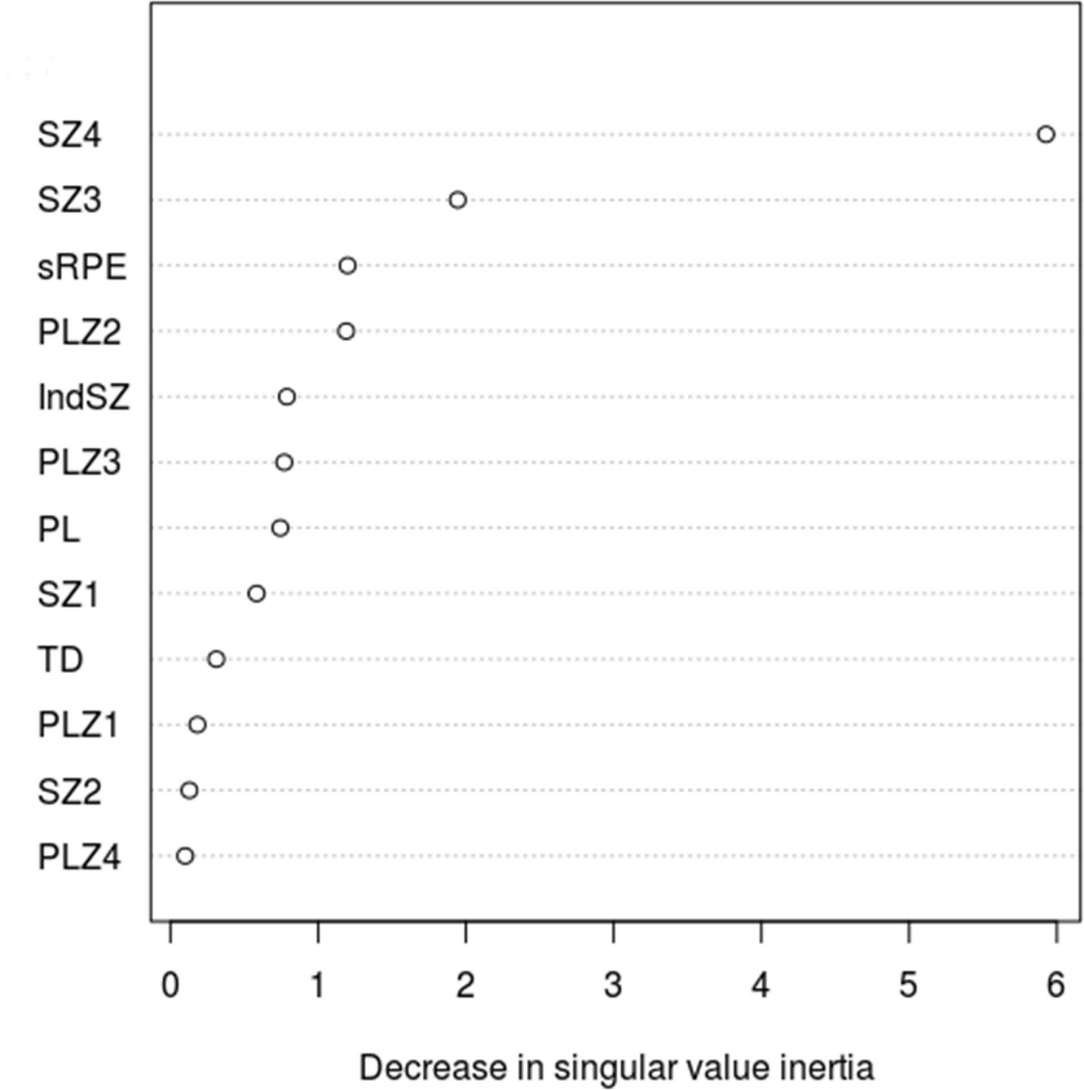
Variable importance plot showing the decrease in singular value inertia attributable to each predictor variable.

The results of the LOVO PLSCA (Table 5) revealed that the greatest decrease in measured (computed) inertia compared with baseline occurred when the variables SZ3 (1.945) and SZ4 (5.926) were omitted from the PLSCA model, indicating that these were the most influential variables, as illustrated by Fig 2. When only these predictor variables were used to construct the PLSCA model (Table 6), the p value achieved was 0.015, indicating that the matrix containing the predictor variables SZ3 and SZ4 shared a considerable amount of information with the output matrix containing the variables ‘starting fitness’ and ‘end fitness’ and that therefore they were likely to be the best predictors of end fitness. Given that ‘starting fitness’ can be treated as a covariate, this implies that SZ3 and SZ4 were likely to be the best predictors of end fitness.

**Table 5:** Results of the LOVO PLSCA showing the effect on singular value inertia of omitting variables one at a time.

PLSCA Model	Response variables	Predictor variables	No. of subjects included	No. of simulations	Measured inertia	Change in inertia compared to baseline	Significance p value
PLSCA Baseline	End Fitness, Starting Fitness	TD, SZ1, SZ2, SZ3, SZ4, PL, sRPE, IndSZ, PLZ1, PLZ2, PLZ3, PLZ4	16	10000	25.096	NA	0.271
Omit TD	End Fitness, Starting Fitness	SZ1, SZ2, SZ3, SZ4, PL, sRPE, IndSZ, PLZ1, PLZ2, PLZ3, PLZ4	16	10000	24.785	0.311	0.240
Omit SZ1	End Fitness, Starting Fitness	TD, SZ2, SZ3, SZ4, PL, sRPE, IndSZ, PLZ1, PLZ2, PLZ3, PLZ4	16	10000	24.512	0.584	0.253
Omit SZ2	End Fitness, Starting Fitness	TD, SZ1, SZ3, SZ4, PL, sRPE, IndSZ, PLZ1, PLZ2, PLZ3, PLZ4	16	10000	24.967	0.129	0.232
Omit SZ3	End Fitness, Starting Fitness	TD, SZ1, SZ2, SZ4, PL, sRPE, IndSZ, PLZ1, PLZ2, PLZ3, PLZ4	16	10000	23.151	1.945	0.774
Omit SZ4	End Fitness, Starting Fitness	TD, SZ1, SZ2, SZ3, PL, sRPE, IndSZ, PLZ1, PLZ2, PLZ3, PLZ4	16	10000	19.170	5.926	0.547
Omit PL	End Fitness, Starting Fitness	TD, SZ1, SZ2, SZ3, SZ4, sRPE, IndSZ, PLZ1, PLZ2, PLZ3, PLZ4	16	10000	24.352	0.744	0.261
Omit sRPE	End Fitness, Starting Fitness	TD, SZ1, SZ2, SZ3, SZ4, PL, IndSZ, PLZ1, PLZ2, PLZ3, PLZ4	16	10000	23.897	1.200	0.273
Omit IndSZ	End Fitness, Starting Fitness	TD, SZ1, SZ2, SZ3, SZ4, PL, sRPE, PLZ1, PLZ2, PLZ3, PLZ4	16	10000	24.308	0.788	0.260
Omit PLZ1	End Fitness, Starting Fitness	TD, SZ1, SZ2, SZ3, SZ4, PL, sRPE, IndSZ, PLZ2, PLZ3, PLZ4, Starting Fitness	16	10000	24.913	0.183	0.233
Omit PLZ2	End Fitness, Starting Fitness	TD, SZ1, SZ2, SZ3, SZ4, PL, sRPE, IndSZ, PLZ1, PLZ3, PLZ4	16	10000	23.906	1.190	0.278
Omit PLZ3	End Fitness, Starting Fitness	TD, SZ1, SZ2, SZ3, SZ4, PL, sRPE, IndSZ, PLZ1, PLZ2, PLZ4	16	10000	24.325	0.771	0.258
Omit PLZ4	End Fitness, Starting Fitness	TD, SZ1, SZ2, SZ3, SZ4, PL, sRPE, IndSZ, PLZ1, PLZ2, PLZ3	16	10000	24.996	0.100	0.224

Abbreviations: TD = total distance (m); SZ1 = speed zone 1 (0 to 3 m·s^-1^; [m]); SZ2 = speed zone 2 (3.1 to 5 m·s^-1^; [m]); SZ3 = speed zone 3 (5.1 to 7 m·s^-1^; [m]); SZ4 = speed zone 1 (> 7.1 m·s^-1^; [m]); PL = PlayerLoad (AU); PLZ1 = PlayerLoad Zone 1 (0 to 1 AU); PLZ2 = PlayerLoad Zone 2 (1.1 to 2 AU); PLZ3 = PlayerLoad Zone 3 (2.1 to 3 AU); PLZ4 = PlayerLoad Zone 4 (> 3.1 AU); sRPE = session-rating-of-perceived-exertion; IndSZ = Individualised speed zone (> 30–15 intermittent fitness test termination speed).

**Table 6:** Results of the PLSCA using refined models.

PLSCA model	Response variables	Predictor variables	No. of subjects included	No. of simulations	Measure inertia	Significance p value	Chi-square (p values) [Cramers V]
PLSCA Baseline	End Fitness, Starting Fitness	TD, SZ1, SZ2, SZ3, SZ4, PL, sRPE, IndSZ, PLZ1, PLZ2, PLZ3, PLZ4	16	10000	25.096	0.271	NA
PLSCA Model 1	End Fitness, Starting Fitness	SZ4	16	10000	13.459	0.007*	709.5 (<0.0001) [0.188]
PLSCA Model 2	End Fitness, Starting Fitness	SZ3, SZ4	16	10000	16.419	0.015*	2030.8 (<0.0001) [0.319]

Abbreviations: TD = total distance (m); SZ1 = speed zone 1 (0 to 3 m·s^-1^; [m]); SZ2 = speed zone 2 (3.1 to 5 m·s^-1^; [m]); SZ3 = speed zone 3 (5.1 to 7 m·s^-1^; [m]); SZ4 = speed zone 1 (> 7.1 m·s^-1^; [m]); PL = PlayerLoad (AU); PLZ1 = PlayerLoad Zone 1 (0 to 1 AU); PLZ2 = PlayerLoad Zone 2 (1.1 to 2 AU); PLZ3 = PlayerLoad Zone 3 (2.1 to 3 AU); PLZ4 = PlayerLoad Zone 4 (> 3.1 AU); sRPE = session-rating-of-perceived-exertion; IndSZ = Individualised speed zone (> 30–15 intermittent fitness test termination speed).

* p values less than 0.05 considered significant for one-tailed test

When the variables SZ3 and SZ4 were subsequently used, together with ‘starting fitness’ as a covariate, to construct linear regression models (Table 7), it was found that the multicollinearity problems disappeared, with all the VIF values being <2.5. Furthermore, the models produced were particularly strong, both exhibiting adjusted r^2^ values >0.7. Of the two models produced, the one containing just the predictor variable SZ4 and ‘starting fitness’ (i.e. MLR Model 1) was the strongest, possessing an Akaike information criterion (AIC) value of 28.3 (lower than the AIC value of 30.1 for MLR Model 2, which also included variable SZ3), indicating that these two variables were the most influential in predicting end fitness, corroborating the results of the LOVO PLSCA.

**Table 7:** Results of refined MLR models with respective variable inflation factors.

MLR model	Response Variable	Predictor Variables	Coefficient b (95% CI)	Standard Coeff. (beta)	Significance (p value)	VIF	Model Adj R^2^	Model p value	AIC
MLR Model 1	End Fitness	Intercept	8.555 (3.172–13.939)	NA	0.004	NA	0.733	<0.001	28.3
		SZ4	0.002 (0.000–0.003)	2.477e-07	0.011	1.35			
		Starting Fitness	0.528 (0.196–0.850)	1.606e-03	0.004	1.35			
MLR Model 2	End Fitness	Intercept	8.303 (2.453–14.153)	NA	0.009	NA	0.714	<0.001	30.1
		SZ3	6.082e-05 (0.000–4.495e-04)	5.879e-08	0.739	1.78			
		SZ4	1.675e-03 (0.000–3.530e-03)	6.820e-06	0.073	2.29			
		Starting Fitness	0.537 (0.207–0.877)	0.515	0.005	1.39			

## Discussion

The overall aim of the study was to evaluate the extent to which PLSCA might be helpful when analysing TL data that exhibited considerable multicollinearity. As such, we wanted to identify the TL variables that best related to 30-15_IFT_ performance in young rugby league players following 6-weeks of training. With respect to this, the specific findings of the current study revealed perhaps unsurprisingly, that ‘starting fitness’ is an important covariate of ‘end fitness’, with a strong positive correlation between the two–something that others have observed [30–31]. The strongest regression model (Table 7; MLR Model 1) suggests that professional youth rugby league players with a lower starting fitness require a lower accumulation of distance at very-high speed (> 7 m·s^-1^) (compared to players with a higher starting fitness) to elicit a comparable incremental improvement in end fitness (e.g. +1 km·h^-1^ in v30-15_IFT_) following 6-weeks of training. This model suggests, for example, that a professional youth rugby league player with a starting v30-15_IFT_ of 17.5 km·h^-1^ would require an accumulation of 350m at very-high speed over 6-weeks to improve their v30-15_IFT_ by 1 km·h^-1^ compared to 1050m for a player with a starting fitness of 20.5 km·h^-1^. As such, this regression model could be used to translate TL data (in conjunction with starting fitness) into practical targets for the applied practitioner working with youth rugby league players. However, it is important to note that this relationship (and associated MLR model) was observed within a single team, meaning the variability between players in the accumulated distances at very-high-speed are specific to the context of the training modalities prescribed by the coaching staff at this club [32]. We therefore recommend that future researchers conduct randomised control trials with appropriate comparator arms in order to consolidate or refute our findings regarding the importance of the interaction between the distance accumulated at very-high speed and a players starting ‘fitness’ to improving prolonged intermittent running ability in team sport athletes.

Consistent with previous research [4–5,8–11], the findings of the current study indicate that variables commonly used to assess TL tend to be highly correlated (Table 3) and thus contain considerable shared information. As such, they violate assumptions regarding multicollinearity (Table 4), which can be problematic when performing MLR analysis [13–14]. While multicollinearity issues can be addressed by removing variables with a ‘high VIF’ value, this has the disadvantage that it is rather piecemeal and involves making subjective decisions regarding VIF exclusion criteria and the variables to be excluded. Alternatively, principal component regression (PCR) can be used [33–34]. However, while PCR is immune to multicollinearity, it has the great disadvantage that it requires the construction of composite predictor variables, which are difficult to interpret. In response to this, we developed the LOVO PLSCA methodology presented above as an alternative for analysing multicollineated data sets. The results of the current study suggest that the LOVO PLSCA strategy is well suited to the analysis of highly correlated sports performance data, suggesting that it might be a useful tool for researchers and practitioners seeking to better understand the factors that influence sports performance.

The LOVO PLSCA approach echoes that of the ‘decrease in Gini impurity’ strategy frequently used with random forests to quantify variable importance [23]. As such, it represents a new orthogonal approach for quantifying variable importance that is immune to multicollinearity and can be used as a variable filtering tool prior to MLR. Furthermore, unlike MLR, which struggles when the number of predictor variables exceeds the number of subjects or observations [35]., PLSCA is not affected by this problem. With PLSCA it is possible to explore the relationship between multiple predictor variables and multiple response variables, enabling complex relationships within the data to be evaluated–something that may be of great value when investigating broad latent constructs such as ‘strength’, ‘fatigue’ or ‘technical-tactical performance’. Say for example, we wanted to evaluate the relationship between, ‘fatigue’ status (measured using the variables: ‘change in countermovement jump height’; ‘perceived recovery’; and ‘6 second watt bike test’) and ‘physical performance’ (measured using the variables: ‘v30-15_IFT_’; ‘40 metre maximal sprint speed’; and ‘3 repetition maximal squat and bench press’). With PLSCA it would be possible to investigate the relationship between the four ‘physical performance’ variables and the three ‘fatigue’ variables, something that would be difficult using a more conventional approach.

Although LOVO PLSCA can be used to assess the relative importance of predictor variables, because it is not a regression technique it cannot explicitly predict the response variables from a set of predictor variables [19]. In order to do this a related technique, partial least squares regression (PLSR), has been developed [19,21]. While consideration of PLSR is beyond the scope of the current paper, it is worth noting that PLSR shares many similarities with PCR in so much that both techniques are primarily used for prediction and require the construction of composite predictor variables, albeit using different methodologies. As such, PLSR suffers from the same drawback as PCR, namely that the models produced are difficult to interpret because the predictors are not the original measured variables. By comparison, PLSCA, when used as a filtering tool and combined with MLR, overcomes any multicollinearity problems and is much easier to interpret.

## Conclusions

The findings of the current study demonstrate that multicollinearity is a major limiting factor, which has the potential to compromise analysis of TL data. However, this problem can be overcome by using an orthogonal PLSCA approach, which is immune to multicollinearity, thus enabling the user to quantify the strength of the relationships between the respective variables. Using LOVO PLSCA we were able to identify those variables that were most influential in explaining improvements in player fitness. This enabled us to remove irrelevant variables and so overcome any multicollinearity issues. This allowed us to produce a robust MLR model for predicting ‘end fitness’, from which we inferred that ‘starting fitness’ and the accumulation of distance at ‘very-high speed’ across a 6-week period of training were the most influential predictors of end fitness in professional youth rugby league players. As such, PLSCA appears to be a useful tool for filtering out irrelevant information and identifying those variables that should be included prior to any given MLR analysis.
